# Supplementary material for: MiR-155-5p Elevated by Ochratoxin A Induces Intestinal Fibrosis and Epithelial-to-Mesenchymal Transition through TGF-β Regulated Signaling Pathway In Vitro and In Vivo
Source: Toxins (Basel). 2023 Jul 22;15(7):473. doi: 10.3390/toxins15070473 (PMC10467050; doi:10.3390/toxins15070473)
Supplement: Supplementary file 1 [file toxins-15-00473-s001.zip › toxins-2452827-supplementary.pdf]

toxins

MDPI

Supplementary Materials: MiR-155-5p Elevated by Ochratoxin A Induces Intestinal Fibrosis and Epithelial-to-Mesenchymal Transition through TGF-β Regulated Signaling Pathway In Vitro and In Vivo

Kyu Hyun Rhee, Seon Ah Yang, Min Cheol Pyo, Jae-Min Lim and Kwang-Won Lee *

Supplementary Table S1. qRT-PCR primer sequences

Origin	Marker		Sequence (5′→3′)
Human	Fibronectin	Forward	CTG GCC GAA AAT ACA TTG TAA A
		Reverse	CCA CAG TCG GGT CAG GAG
	α-SMA	Forward	CTC TCT GTC CAC CTT CCA G
		Reverse	TAA CGA GTC AGA GCT TTC GC
	E-cadherin	Forward	GCC TCC TGA AAA GAG AGT GGA AG
		Reverse	TGG CAG TGT CTC TCC AAA TCC G
	TGF-β	Forward	CAA CAA TTC CTG GCG ATA CC
		Reverse	GCT AAG GCG AAA GCC CTC AAT
	C/EBPβ	Forward	CTT CAG CCC GTA CCT GGA G
		Reverse	GGA GAG GAA GTC GTG GTG C
	GAPDH	Forward	TGC ACC ACC AAC TGC TTA GC
		Reverse	GGC ATG GAC TGT GGT CAT GAG
Mouse	Fibronectin	Forward	CAC GAT GCG GGT CAC TTG
		Reverse	CTG CAA CGT CCT CAT TCT TC
	α-SMA	Forward	TCC TGA CGC TGA ACT ATC CG
		Reverse	GGC CAC ACG AAG CTC CTT AT
	E-cadherin	Forward	CCA AGC AGC AGT ACA TTC TAC A
		Reverse	CAT TCA CAT CCA GCA CAT CCA
	TGF-β	Forward	CAC CGG AGA GCC CTG GAT A
		Reverse	TGT ACA GCT GCC GCA CAC A
	C/EBPβ	Forward	ATC GAC TTC AGC CCC TAC CT
		Reverse	TAG TCG TCG GCG AAG AGG
	GAPDH	Forward	AGA ACA TCA TCA TCC CTG CAT CCA
		Reverse	CCG TTC AGC TCT GGG ATC AC
